# Zika virus infection

**DOI:** 10.1590/0100-3984.2019.52.6e3

**Published:** 2019

**Authors:** Heron Werner Jr.

**Affiliations:** 1 Fetal Medicine Specialist - Clínica Alta Excelência Diagnóstica/DASA, Rio de Janeiro, RJ, Brazil. E-mail: heron.werner@gmail.com; heronwerner@hotmail.com

Zika virus (ZIKV) is an arbovirus composed of RNA and belonging to the family Flaviviridae (genus *Flavivirus*), the same family that includes the dengue and chikungunya viruses. Transmission is by the *Aedes* mosquito and shows a potential association with neurological and autoimmune complications such as congenital microcephaly, adult paralysis disorder and Guillain-Barré syndrome. Infection with ZIKV is self-limiting, and only 20% of infected patients develop symptoms, which persist for 5-7 days and are usually mild in nature, including fever, joint pain, myalgia, maculopapular rash, retro-orbital headache and conjunctivitis^(1-3)^.

From its discovery in 1947 in monkeys of Uganda and the first human ZIKV infection, reported in Nigeria five years later, until its arrival in South America, it was not known that the virus would be capable of having marked effects. However, nearly 50 years passed before there were a significant number of infections, the first outbreak being reported in 2007 on the Micronesian island of Yap. Epidemic spread of the disease was also observed in the South Pacific-in French Polynesia in 2013 and in New Caledonia in 2014. Infection with ZIKV occurs in tropical and subtropical areas, and there are two strains (Asian and African), originating from a common ancestor^(1,4)^.

Like other arboviruses, ZIKV participates in a complex transmission cycle between primates and mosquitoes, in which humans are occasional, unintended hosts. The vector of transmission is the *Aedes aegypti* mosquito, which also transmits yellow fever, dengue, and chikungunya. The pace of urbanization in recent decades has led to the accumulation of millions of inhabitants in various cities. The level of social vulnerability in such cities may have contributed to the increase in the number of cases of ZIKV infection. The *A. aegypti* mosquito adapted easily to the urban environment, due to the high human population density and the great number of artificial breeding sites such as standing water and piles of garbage.

In 2011, another probable form of transmission, sexual transmission, was reported. Therefore, the known routes of transmission are transplacental, by blood transfusion, and by sexual contact^(5)^.

Laboratory diagnosis of the infection is based on identification of the virus in the blood (acute phase) and urine (after the first week of symptoms) with reverse-transcriptase polymerase chain reaction. Viral RNA can also be identified in amniotic fluid and cerebrospinal fluid. Serological tests for anti-ZIKV immunoglobulin M antibodies can also be used on the 4th or 5th day after symptom onset. Such antibodies can remain detectable for 2-3 months, as is the case of other flaviviruses. However, they are not specific to ZIKV. Cross-reactions with other flaviviruses are quite common and make diagnosis impossible in individuals who have a history of infection with viruses such as dengue and chikungunya or have been vaccinated against yellow fever^(3,5)^.

In 2016, it was recognized internationally that ZIKV infection during pregnancy could cause fetal malformations, including microcephaly. However, the magnitude of the risk of such malformations has yet to be clearly defined^(6)^. In Latin America, Brazil was the most affected country, the first case being reported in the state of Bahia in 2015. There was a sharp increase in the number of cases of microcephaly from March 2015 to February 2016^(7-9)^.

Neural progenitor cells are the primary target of ZIKV, which explains the great number of fetal central nervous system (CNS) changes seen on neuroimaging examinations^(10)^. It is now known that ZIKV-related CNS damage occurs in multiple ways, microcephaly being considered the tip of the iceberg, because it actually represents the epilogue of a devastating process of the infection in the fetal CNS^(8)^. Although neuroimaging findings in congenital ZIKV syndrome are not pathognomonic, many are quite suggestive of the diagnosis; the radiologist should therefore be prepared to recognize and interpret such features, as well as to suggest the diagnosis^(3,11)^.

Fetal changes resulting from intrauterine ZIKV infection are more severe when they occur in the first or second trimesters of pregnancy, such changes ranging from fetal death to various congenital anomalies such as redundant neck skin with occipital bone proeminence, low birth weight, anasarca, arthrogryposis, hearing loss, polyhydramnios, ocular malformations, and CNS anomalies^(3,12)^. The fetal abnormalities most commonly visualized by ultrasound and magnetic resonance imaging (MRI) are microcephaly, ventriculomegaly, and multifocal calcifications, less common abnormalities including posterior fossa alterations, such as cerebellar and pontine hypoplasia^(3,11)^. In the postnatal period, the main lesions are visualized by ultrasound, computed tomography (CT), or MRI. Perinatal MRI and CT allow the diagnosis of pachygyria, dysgenesis of the corpus callosum, cortical atrophy, and a small anterior fontanelle with premature closure of the cranial sutures^(12-16)^.

In cases of ZIKV infection, the only therapeutic option is symptomatic treatment. The main focus is on prevention measures, such as eliminating the vector and limiting travel to endemic areas^(3)^.
